# Kinetic Uptake Studies of Powdered Materials in Solution

**DOI:** 10.3390/nano5020969

**Published:** 2015-06-04

**Authors:** Mohamed H. Mohamed, Lee D. Wilson

**Affiliations:** Department of Chemistry, University of Saskatchewan, 110 Science Place, Saskatoon S7N 5C9, Canada; E-Mail: mohamed.mohamed@usask.ca

**Keywords:** kinetics, dye adsorption, cyclodextrin, polyurethane, *p*-nitrophenol, phenolphthalein, nanomaterials

## Abstract

Challenges exist for the study of time dependent sorption processes for heterogeneous systems, especially in the case of dispersed nanomaterials in solvents or solutions because they are not well suited to conventional batch kinetic experiments. In this study, a comparison of batch versus a one-pot setup in two variable configurations was evaluated for the study of uptake kinetics in heterogeneous (solid/solution) systems: (i) conventional batch method; (ii) one-pot system with dispersed adsorbent in solution with a semi-permeable barrier (filter paper or dialysis tubing) for *in situ* sampling; and (iii) one-pot system with an adsorbent confined in a semi-permeable barrier (dialysis tubing or filter paper barrier) with *ex situ* sampling. The sorbent systems evaluated herein include several cyclodextrin-based polyurethane materials with two types of phenolic dyes: *p*-nitrophenol and phenolphthalein. The one-pot kinetics method with *in situ* (Method ii) or *ex situ* (Method iii) sampling described herein offers significant advantages for the study of heterogeneous sorption kinetics of highly dispersed sorbent materials with particles sizes across a range of dimensions from the micron to nanometer scale. The method described herein will contribute positively to the development of advanced studies for heterogeneous sorption processes where an assessment of the relative uptake properties is required at different experimental conditions. The results of this study will be advantageous for the study of nanomaterials with significant benefits over batch kinetic studies for a wide range of heterogeneous sorption processes.

## 1. Introduction

The study of conventional kinetic uptake experiments for two phase (e.g., solid-solution) systems is often met with several experimental challenges [1,2,3]. In particular, powdered adsorbent materials are generally studied using batch kinetic experiments. The time dependence of the adsorption profile relies on rapid phase separation of components which require work-up procedures after the kinetic uptake process to allow for reliable temporal sampling of the solution phase composition. Moreover, discrepancies can arise from inadequate separation of phases when centrifugation of supernatant is used to carry out phase separation of a two-phase (e.g., solid-solution) system to quantify the solution phase composition [4]. Similar challenges exist for the use of filter media such as membranes [5] for the separation of two-phase systems. The use of filtration media is problematic for quantitative analysis, especially when competitive adsorption occurs onto the media when filtering the solid-solution components. An additional challenge concerning the rapid separation of multi-phase systems occurs when employing batch methods for the study of uptake kinetics at variable temperature. By contrast, one-pot sorption experiments have been employed which rely on sampling of aliquots in a multi-phase system as a function of time. This method often neglects the loss of adsorbent during sampling of heterogeneous fractions which contain the sorbent phase residues that are often unaccounted for during the serial sampling process [6]. In either case, the utility of batch experiment studies relies on well-defined differences in the time resolution of the sampling interval relative to the kinetics of the adsorption process [7]. Batch kinetics of multiple samples can overcome these challenges but the method is time consuming, labor-intensive, requires substantive amounts of sorbent material, and may be inapplicable to solid-solution systems with rapid uptake kinetics due to the aforementioned systematic errors over the sampling interval [2].

To address the shortcomings of conventional batch kinetics for heterogeneous systems, an alternative approach is required, especially in the case of colloidal nanomaterials which cannot be readily separated by routine centrifugation or filtration methods, as described above. Herein, we report a comparative study of conventional batch kinetics that uses a facile one-pot method with two different sampling configurations (*i.e.*, *in situ* *vs.* *ex situ*). The one-pot method relies on physical separation of the solid phase and the solution with adsorbate species using a semi-permeable barrier [8]. The study herein employs two types of model phenolic dyes (*p*-nitrophenol; PNP and phenolphthalein; phth) in aqueous solution with several types of cyclodextrin-based polyurethane materials (PUs) as the adsorbent phase for evaluation of the uptake properties at variable kinetic conditions. In a previous study [9], various types polymer sorbent/dye systems were characterized at equilibrium conditions using batch conditions and provide a useful reference to compare the kinetic uptake results. The results of this study examine the utility of the one-pot kinetic method for the study of the sorptive uptake for solid-solution systems. The results are compared with the uptake kinetics of the same systems using conventional batch methods for solid-solution systems. The one-pot method may employ *in situ* or *ex situ* sampling and offers several advantages over conventional batch methods. The one-pot method described herein employs reduced amounts of sorbent material and is amenable to studies at variable temperature, and is applicable to highly dispersed and colloidal sorbents such as nanomaterials that often present experimental challenges by conventional batch methods, as described above.

## 2. Results and Discussion

**Scheme 1:**
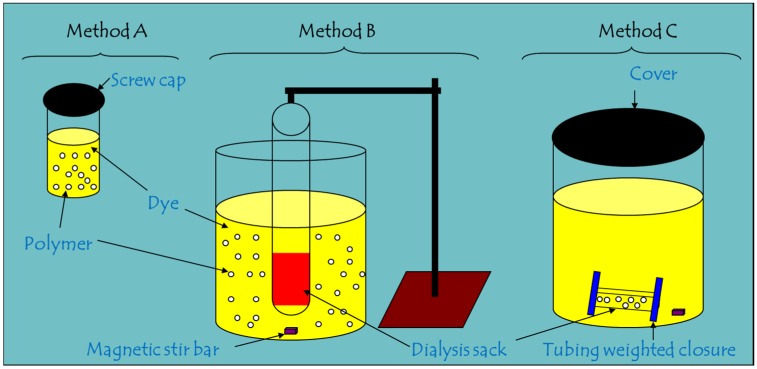
Three experimental configurations for the study of solid-solution adsorption kinetics. **Method A**; conventional batch kinetics, **Method B**; dispersed adsorbent with *in situ* sampling of adsorbate phase, and **Method C**; confined adsorbent in a membrane (or filter paper) with *ex situ* sampling of the adsorbate phase.

As indicated above, there are challenges for the study of adsorption processes of powdered materials depending on their relative particle size when using conventional batch methods. Herein, a series of insoluble polymer materials and phenolic dyes in solution were selected to study conventional batch kinetics using a facile one-pot method that employs two different sampling configurations (*i.e.*, *in situ vs. ex situ*). The polymer-dye systems were chosen since the uptake mechanism at equilibrium conditions was reported elsewhere [9]. To evaluate the uptake properties of materials in the form of insoluble powdered materials, a comparison of conventional batch kinetics (Method A) was compared against a one-pot setup with two different configurations (Methods B and C), as illustrated in Scheme 1. Batch kinetic methods (Method A) have been discussed in detail elsewhere [6,7]; whereas, the one-pot method employed herein uses a barrier material with variable pore size (*i.e.*, dialysis membrane or filter paper) which allows quantitative assessment of the concentration of unbound adsorbate species by *in situ* sampling (Method B) *vs. ex situ* sampling (Method C). It should be noted that there are systematic errors when using such one-pot methods without a barrier material due to the inadvertent removal of sorbent material during sampling of aliquot volumes for subsequent quantitative analyses, especially spectroscopic analyses. It will be shown below that the use of the one-pot setup (*i.e.*, Methods B or C) offers significant advantages for the study of uptake kinetics for heterogeneous sorption phenomena in solid-solution systems for a diverse range of adsorbent materials.

### 2.1. Dye Uptake Kinetics Equations and Models

The temporal dependence of the isotherms are represented as plots of the amount of dye adsorbed per mass of polymer material (*Q_t_*) *vs.* time (*t*) where *Q_t_* is defined by Equation (1). The value of *Q_t_* is defined by Equation (1) where *C_o_* is the initial adsorbate (dye) concentration, *C_t_* is the dye concentration at any time (*t*), *V* is the volume of solution, and *m* is the mass of sorbent.

(1) $${\text{Q}}_{\text{t}}=\frac{({\text{C}}_{\text{o}}-{\text{C}}_{\text{t}})\times \text{V}}{\text{m}}$$

The data was fit with a pseudo-first order (PFO) [10] or a pseudo-second order (PSO) [11] model, according to Equations (2) and (3), respectively. *Q_t_* and *Q_e_* are the levels of dye adsorbed at any particular time (*t*) and at pseudo-equilibrium, respectively. *k_i_* is a rate constant (*i* = 1 (*k*_1_) for the PFO and *i* = 2 (*k*_2_) for the PSO models, respectively) where *t* represents time. The sum of square of errors (SSE) was used as a criterion of the “best fit” where a lower value of SSE (*cf*. Equation (4)) indicates an overall “best-fit”. Consideration of the mechanism of dye uptake was made since it is known that phenolphthalein (phth) is bound at the CD inclusion sites; whereas, *p*-nitrophenol (PNP) is bound at the inclusion sites along with the cross-linker domains [9]. This was obtained by minimization of the SSE for all data across the range of experimental conditions. Thus, single site and dual site binding are considered when choosing the appropriate kinetic models (Equations (2) and (3)) along with the relative difference in SSE values obtained by Equation (4). *Q_ti_* is the experimental value, *Q_ef_* is the fitted value and *N* is the number of *Q_t_* data points:

(2) $${\text{Q}}_{\text{t}}={\text{Q}}_{\text{e}}(1-{\text{e}}^{-{\text{k}}_{\text{1}}\text{t}})$$

(3) $${\text{Q}}_{\text{t}}=\frac{{{\text{Q}}_{\text{e}}}^{2}{\text{k}}_{2}\text{t}}{1+{\text{k}}_{2}{\text{tQ}}_{\text{e}}}$$

(4) $$SSE=\sqrt{\frac{{\left(Q_{ti}-Q_{ef}\right)}^{2}}{N}}$$

### 2.2. Comparison of Batch and One-pot Kinetic Methods

**Figure 1:**
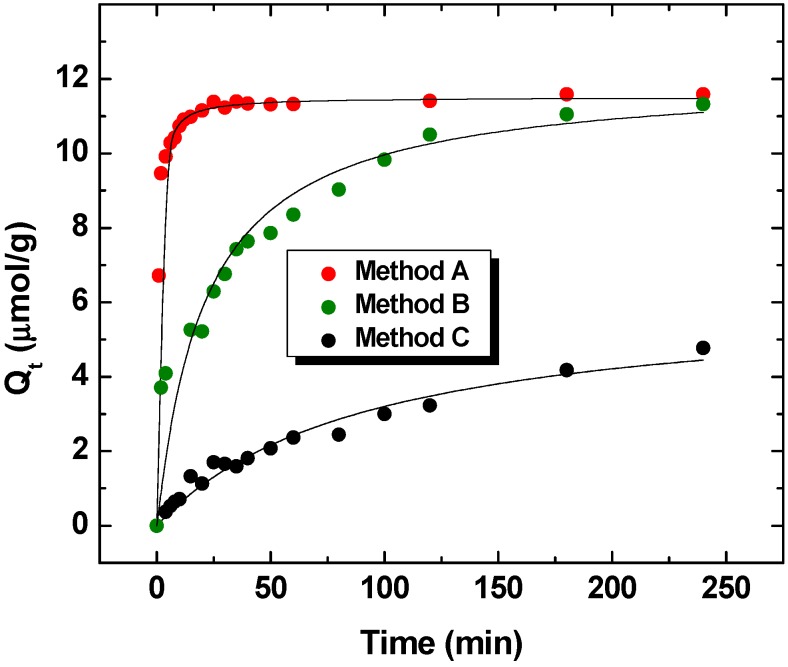
Kinetic profiles for a cross-linked organic copolymer (CDI-2) with *p*-nitrophenol (PNP) in aqueous solution at pH 4.60 and 298 K using different experimental methods. The solid lines represent the “best-fit” using the pseudo-second order (PSO) kinetic model.

Figure 1 illustrates the time dependent uptake of CDI-2 with PNP according to the batch and one-pot methods (A–C; *cf*. Scheme 1). The relative rate of dye uptake varies according to each method as follows: Method A > Method B > Method C. The time dependence of the adsorption profile for each system is well described by the PSO model as evidenced by the “best-fit” results in Figure 1. The uptake of PNP is consistent with dual binding sites reported for such urethane copolymers according to a previous equilibrium study [9]. Dual adsorption sites are represented by the β-CD inclusion sites and the interstitial cross-linker domains of the polymer framework [9]. It should be noted that the relative binding affinity of the inclusion sites generally exceed that for the interstitial domains [K(inclusion) > K(interstitial)] due to differences in the preorganization of each binding site [12]. The value of *Q_e_* (µmol/g) varies according to each method (listed in parentheses); 11.5 (A), 11.6 (B) and 6.15 (C), and the value for *k*_2_ (g/*µ*mol.min) also varies; 0.139 (A), 0.00458 (B), and 0.00176 (C). Methods A and B yield similar estimates of uptake (*Q_e_*) at equilibrium conditions which agree with results from a previously reported study [9]. However, the rate of dye uptake differs for the one-pot method that uses dialysis tubing since a reduction in the rate constant (*ca*. 30-fold) is observed when comparing *k*_2_ values for Methods A (batch) and B that used dialysis membrane. Method A reaches equilibrium rapidly within 30 min while method B requires a longer equilibration time (~240 min), in agreement with previous observations [13]. The results indicate that dispersion of the insoluble polymer by Method A enables attainment of a maximum value of *Q_e_* over a relatively short time interval that compares well with estimates from conventional batch studies. However, the rate of adsorption is attenuated due to attenuated diffusion across the dialysis membrane when using the one-pot system (Method B). An advantage of Method B over Method A involves the direct sampling and analysis of the isolated aliquots which contain unbound adsorbate since the barrier material enables *in situ* separation of any measurable amounts of adsorbent. Sampling of a homogeneous (single phase) solution affords direct and reliable quantitation of the unbound adsorbate with good time resolution without the need for further separation of phases. By contrast, conventional batch methods require centrifugation or filtration to separate sorbent and solution phases. One drawback of method B is that sorbent materials may tend to locate at interfaces (e.g., air/water, glass/solution, membrane/solution) when the density of the material is low. Thus, the nonuniform dispersion of powdered materials may occur for such solid-solution systems; thereby, limiting an accurate assessment of the solid-solution uptake kinetics. The one-pot Method C may offer an improvement over Method B due to confinement of the sorbent in a fixed volume within the barrier material (*cf*. Scheme 1). However, the value of *Q_e_* is ~ two-fold lower for Method C relative to Methods A and B. The attenuated value of *Q_e_* for Method C is related to a reduction in mass transfer due to confinement the sorbent within a smaller volume. Such differences in the uptake kinetic processes for Methods B and C, the results are consistent with attenuated rates of diffusion and variable mass transfer between the bulk and adsorbent phases [14]. The foregoing is consistent with the lower value of *k*_2_ obtained by Method C since it is *ca*. eighty-fold lower than Method A. *Q_t_* was measured after 24 h and found to be 9.58 µmol/g. The value of *Q_e_* obtained from Method C converges with the results obtained from Methods A and B at sufficiently long time intervals (24 h) as the isotherm approaches saturation, in agreement with the above discussion. Although Methods B and C may not provide absolute estimates of kinetic rate parameters (*Q_t_* and *k_i_*) due to the mass transfer effects of the adsorbate across the barrier material (dialysis membrane or filter paper), Methods B and C offer a convenient approach for the systematic comparison of different adsorbent materials on a relative scale. At controlled experimental conditions, Methods B and C enable the study of colloidal materials using the one-pot set up with *in situ* or *ex situ* sampling with an appropriate choice of barrier material. Batch methods do not allow for the reliable study of colloidal nanomaterials due to less technical challenges when separating the solid and solution phases. The ability to estimate the concentration of unbound adsorbate for relatively rapid kinetic uptake processes with a one-pot system is feasible using Methods B or C. As well, the one-pot system is suitable for maintaining temperature control for variable temperature studies due to the advantage of direct sampling of unbound adsorbate species using Methods B or C. The one-pot method can be scaled to use minimal amounts of adsorbent when compared with conventional batch adsorption studies [8]. As well, the one-pot system using Methods B and C attenuate the “apparent” uptake kinetics or increase the time interval required to achieve saturation of the isotherm due to diffusion and mass transfer effects, as described above. The attenuation of the uptake kinetics across the barrier results in a longer time interval relative to conventional batch methods. The one-pot system (Method C) bears a close similarity to the stir bar sorptive extraction (SBSE) method [15,16]; however, the use of powdered materials offers significant advantages. The SBSE method requires preparation of a well-defined polymer film that is resistant to mechanical damage whilst stirring. The preparation of well-defined polymer films in the case of SBSE studies may present challenges for quantitative analysis as compared with the one-pot method that can utilize diverse powdered sorbent materials with variable morphology due to the use of the semi-permeable barrier.

### 2.3. Comparison of Sorbent Materials with Phenolic Dyes

**Figure 2:**
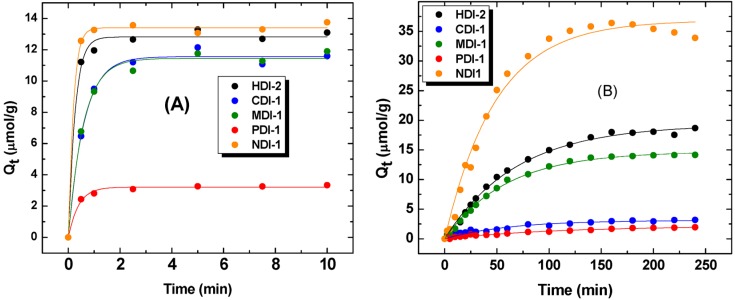
Kinetic uptake isotherms for polyurethane materials (PUs) with phth at pH 10.5 and 25 °C for; (**A**) Method A, and (**B**) Method C. The solid lines represent the “best-fit” using the pseudo-first order (PFO) kinetic model.

Methods A and C were further studied by comparison of a series of PUs with different structural forms [17] and their uptake properties with phth at pH 10.5 (*cf*. Figure 2). Unlike the kinetic uptake studies of PNP above, the barrier material used for phth was a cellulose-based filter paper instead of a dialysis membrane. The observed difference in mass transfer between different barrier materials arises because of changes in the barrier pore-size and the zeta-potential at various pH conditions. In the case of dialysis tubing, the mass transfer was observed to be highly attenuated in the case of PNP at pH 9. Therefore, the uptake kinetics was studied at conditions where charge repulsion was minimized (pH 4.6). Zeta-potential effects were less pronounced in the case of filter paper due to its relatively constant zeta-potential over a wide pH range. Thus, the uptake kinetic experiments at alkaline conditions with conventional filter paper as the barrier medium provides reliable results due to the faster diffusion processes. The PFO model provided the “best-fit” for the adsorptive processes where the values of *Q_e_* and *k_t_* derived from the PFO model are listed in Table 1. The batch process (Method A) shows that the *Q_e_* values decrease as follows: NDI-1 > HDI-2 > CDI-1 ≈ MDI-1 > PDI-1. Method C reveals the following trend in *Q_e_* values: NDI-1 > HDI-2 > MDI-1 > CDI-1 > PDI-1. Each respective method reveals self-consistent results except for the CDI-1 material. The trends for *k*_1_ are not readily comparable since the relative uptake for a given polymer is highly dependent on fractional coverage of the adsorbent surface. Methods B and C employ different dosage of sorbent (sorbent weight per given solution volume). Method C is versatile and provides a valuable comparison of the adsorptive properties of the PUs with phth due to the enhanced uptake profile, as shown by a 25-fold difference relative to method B for the reasons described above.

**Table 1:** *Q_e_* and *k*_1_ values obtained from pseudo-first order (PFO) kinetic model using the data obtained in Figure 2.

Copolymer	Method A		Method C	
	*Q_e_* (μmol/g)	*k*_1_ (s^−1^)	*Q_e_* (μmol/g)	*k*_1_ (s^−1^)
HDI-2	12.8	237	19.2	0.875
CDI-1	11.6	100	3.13	0.978
MDI-1	11.4	103	14.7	1.02
PDI-1	3.21	156	2.17	0.54
NDI-1	13.4	330	36.9	1.23

### 2.4. Comparison of Sorbent Materials with Phenolic Dyes at Variable Temperature

**Figure 3:**
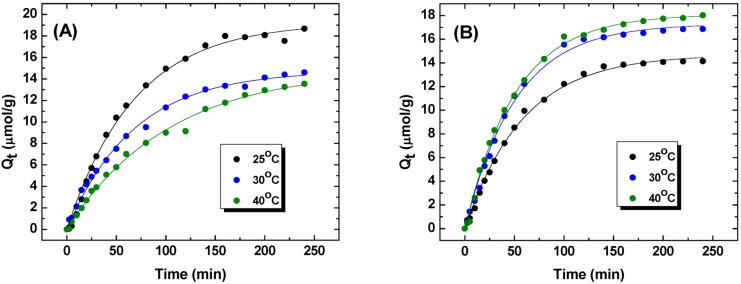
Kinetic uptake profiles for PUs with phth at pH 10.5 and variable temperatures (25, 30 and 40 °C) using Method C for; (**A**) HDI-2 and (**B**) MDI-1. The solid lines represent “best-fit” using a pseudo-first order (PFO) kinetic model.

The kinetic profiles of HDI-2 and MDI-1 polymers at variable temperature are shown in Figure 3. The results reveal measurable differences in values of *Q_e_* and *k*_1_ for each material with increasing temperature (*cf*. Table 2). In general, many adsorptive processes are enthalpy driven [18] if one ignores entropic contributions that arise due to hydration or solvation phenomena. The results for HDI-2 in Figure 3a represent a decrease in sorptive uptake with increasing temperature which indicates that the sorptive process is driven exothermally. By contrast, an opposite temperature dependence of kinetic uptake is observed for MDI-1. The main difference between these polymers is the nature of the linker and the level of cross-linking for HDI-2. HDI is a flexible aliphatic linker, whereas; MDI is more rigid in nature due to the presence of aromatic ring moieties. The greater cross-linking of HDI-2 may offset any differences in the flexibility of each system. The observed differences for HDI-2 and MDI-1 according to the positive and negative temperature dependence of *Q_t_* may relate to differences attributed to hydration and/or steric effects since the values of *Q_e_* are generally comparable for each system. The uptake values at equilibrium conditions using batch conditions were reported elsewhere for these polymer systems where it was concluded that steric effects arising from cross-linking govern the sorptive uptake of phth for these CD polymer/dye systems [19,20]. Notwithstanding the structural differences between cross-linkers and their relative composition, the use of the one-pot method enables the study of structurally related materials where variable uptake properties can be detected that are often difficult to measure by conventional batch techniques. Detailed studies of this type are anticipated to provide insight about the sorption mechanism of structurally related polymer materials. It is noteworthy that the kinetic models of adsorption for PUs with PNP and phth are the PSO and PFO, respectively. The PSO model is understood due to the fact that PNP is bound at the inclusion and non-inclusion sites of the polymer [9]. By contrast, the PFO model describes the uptake of phth since it is bound mainly at the inclusion sites of β-CD in the polymer framework [9,19]. Therefore, the best-fit of the kinetic uptake results agree with previous results obtained at equilibrium conditions that show either one-site (PFO model) or dual site binding (PSO model), respectively.

**Table 2:** *Q_e_* and *k*_1_ values obtained from PFO kinetic model using the data obtained in Figure 3.

Temperature	HDI-2		MDI-1	
	*Q_e_* (μmol/g)	*k*_1_ (s^−1^)	*Q_e_* (μmol/g)	*k*_1_ (s^−1^)
25 °C	19.2	0.875	14.7	1.02
30 °C	14.8	0.886	17.2	1.17
40 °C	14.9	0.581	18.1	1.18

## 3. Experimental Section

### 3.1. Materials

The PUs were synthesized by crosslinking β-cyclodextrin with various cross-linkers, *i.e.*, 1,6-hexamethylene diisocyanate (HDI), 4,4'-dicyclohexylmethane diisocyanate (CDI), 4,4'-diphenylmethane diisocyanate (MDI), 1,4-phenylene diisocyanate (PDI) and 1,5-naphthalene diisocyanate (NDI). The nomenclature of the PUs is described according to the type of diisocyanate and the relative reagent mole ratio (β-cyclodextrin: diisocyanate linker). Details of the synthesis and characterization was reported elsewhere [17]. PNP and phth were used as model phenolic adsorbates in this kinetic study due to their differences in size, solubility, and lipophilic surface area. Stock solutions were serially diluted to prepare dye solutions with variable concentration. 1.0 × 10^−4^ M PNP was prepared at pH 4.60 in 0.01 M potassium phosphate monobasic buffer solution. 4.2 × 10^−5^ M phth was prepared from a stock solution of phth in ethanol [19].

### 3.2. Kinetic Uptake Methods

Three kinetic methods (A, B, and C; see Scheme 1) were examined to evaluate the uptake of PNP with various sorbent materials, as described below. Methods A and B were used to study PNP at various conditions while Method C was used to evaluate uptake of phth at various temperatures.

**Method A:** 10 mg of the PUs was weighed in several 2 dram vials. 7.0 mL of dye solution was added, covered with parafilm before sealing with a screw-cap lid. The vials was shaken in a horizontal shaker (150 rpm) for variable time periods and stopped to measure uptake at variable time. The copolymer was immediately separated from the solution phase by briefly centrifuging for 2 min and the supernatant was quantitatively analyzed using UV-vis spectrophotometry with a double beam spectrophotometer (Varian CARY 100, Agilent Technologies, Santa Clara, CA, USA) at room temperature (295 ± 0.5 K) by monitoring the absorbance changes of the unbound dye, where λ_max_ = 317 nm (PNP) and λ_max_ =552 nm (phth).

**Method B:** The mid-section of a 60 mL syringe, without the plunger, was cut open (not completely, two opposite sides of ~5 mm in width was left) and wrapped with the dialysis tubing cellulose membrane (average flat width 76 mm (3.0 inch), MWCO 12,000-14,000 Da) using parafilm. The end of the syringe body where the needle was attached was cut and the hole plugged with parafilm. The syringe with the attached dialysis tubing was soaked in the PNP solution for at least 2 h prior to running the kinetic uptake experiment. This was done to hydrate and saturate the adsorption sites of the cellulose membrane (dialysis tubing) since uptake of dye by the barrier material may represent a small contribution to the overall adsorption that can be accounted for by soaking the membrane prior to the kinetic uptake experiment. Pre-saturating the “blank” membrane (no sorbent) accounts for small errors in uptake that are otherwise unaccounted for by the one-pot configuration shown in Scheme 1. To conduct the experiment, the syringe was immersed (held with a clamp on a stand) in a 150 mL beaker containing 100 mL of the PNP solution. 100 mg of the polymer was added and stirred with a magnetic stir bar set at 600 rpm. Three milliliter aliquots of the PNP solution was pipetted out after each designated time interval and analyzed, as outlined for Method A.

**Method C:** Approximately 6 cm of the dialysis tubing cellulose membrane (average flat width 35 mm (1.4 inch), MWCO 12,000 Da) was cut to size and soaked in the aqueous PNP solution for ~2 h. One end of the tubing was clamped using the weighted plastic closures. 100 mg of the polymer was added and the other end was similarly closed. The tubing containing the polymer was immersed in a fixed volume (120 mL) of PNP solution of a known concentration. Three milliliter aliquots of the PNP solution was pipetted out after each designated time interval and quantified by UV-vis absorption. The *ex situ* sampling method was used for monitoring the kinetics of sorption for the various polymer materials with phth. Ahlstrom filter paper (Grade 613, 7.5 cm) was used instead of the dialysis tubing as the barrier material. Stainless steel wire was used to wrap the filter paper to contain the sorbent within the barrier material.

## 4. Conclusions

Herein, we report a simplified experimental design for kinetic uptake studies of heterogeneous solid-solution systems that employ a “one-pot” method with *in situ* or *ex situ* sampling. The “one-pot” method described herein enables time resolved kinetic uptake studies of powdered solids in solution with no subsequent phase separation of the solid sorbent and solution phase. The method is facile and offers some advantages relative to uptake studies that employ conventional batch kinetic methods. The one-pot method differs from conventional batch methods due to mass transfer effects across a semi-permeable barrier. A key advantage of the “one-pot” method for kinetic uptake studies enables direct analysis (*in situ* or *ex situ*) of the unbound adsorbate species. The one-pot method is a versatile method for comparison of the uptake kinetics for structurally related sorbent materials [21,22]. In contrast with conventional batch kinetic uptake studies for solid-solution systems, the challenges for the study of highly dispersed solids, colloids, and nanomaterials can be overcome using the one-pot approach described herein can be applied to adsorption processes at variable temperature conditions.
